# A Trisubstituted Benzimidazole Cell Division Inhibitor with Efficacy against *Mycobacterium tuberculosis*

**DOI:** 10.1371/journal.pone.0093953

**Published:** 2014-04-15

**Authors:** Susan E. Knudson, Divya Awasthi, Kunal Kumar, Alexandra Carreau, Laurent Goullieux, Sophie Lagrange, Hélèn Vermet, Iwao Ojima, Richard A. Slayden

**Affiliations:** 1 Mycobacteria Research Laboratories, Department Microbiology, Immunology and Pathology, Colorado State University, Fort Collins, Colorado, United States of America; 2 Department of Chemistry, Stony Brook University, Stony Brook, New York, United States of America; 3 Institute of Chemical Biology & Drug Discovery, Stony Brook University, Stony Brook, New York, United States of America; 4 Infectious Disease Therapeutic Strategic Unit, Sanofi-Aventis R&D, Toulouse, France; 5 Infectious Disease Therapeutic Strategic Unit, Sanofi-Aventis R&D, Montpellier, France; Colorado State University, United States of America

## Abstract

Trisubstituted benzimidazoles have demonstrated potency against Gram-positive and Gram-negative bacterial pathogens. Previously, a library of novel trisubstituted benzimidazoles was constructed for high throughput screening, and compounds were identified that exhibited potency against *M. tuberculosis* H37Rv and clinical isolates, and were not toxic to Vero cells. A new series of 2-cyclohexyl-5-acylamino-6-*N*, *N*-dimethylaminobenzimidazoles derivatives has been developed based on SAR studies. Screening identified compounds with potency against *M. tuberculosis.* A lead compound from this series, SB-P17G-A20, was discovered to have an MIC of 0.16 µg/mL and demonstrated efficacy in the TB murine acute model of infection based on the reduction of bacterial load in the lungs and spleen by 1.73±0.24 Log_10_ CFU and 2.68±Log_10_ CFU, respectively, when delivered at 50 mg/kg by intraperitoneal injection (IP) twice daily (bid). The activity of SB-P17G-A20 was determined to be concentration dependent and to have excellent stability in mouse and human plasma, and liver microsomes. Together, these studies demonstrate that SB-P17G-A20 has potency against *M. tuberculosis* clinical strains with varying susceptibility and efficacy in animal models of infection, and that trisubstituted benzimidazoles continue to be a platform for the development of novel inhibitors with efficacy.

## Background

Tuberculosis (TB) infects a significant portion of people worldwide resulting in the leading cause of death globally from a bacterial infection. Not all TB infections lead to active disease and in fact latent infections provide an on going source of infection. Despite continued efforts from the research community and clinicians, this reservoir of infection [1], [2] hinders disease management efforts. Current treatment requires 6–9 months therapy with a combination of drugs; 4 drugs for 2–4 months and 2 drugs for an additional 4–5 months, and latent infections are treated with isoniazid for 6–9 months. Contributing factors hindering disease management is the aging anti-tubercular first and second line drugs, which were all discovered more than 4 decades ago, with the discovery of isoniazid in 1951, pyrazinamide in 1952, rifampin in 1957 and ethambutol in 1962, except for the very recent entry of bedaquilline in 2012, and the emergence of multi-drug resistant clinical TB strains (MDR, XDR-TB, TDR-TB). Further, there are concerns that the existing drug regimens even when used appropriately do not result in durable cure. Therefore, the development of next generation chemotherapeutics with a novel mode of action that can be readily incorporated into current drug regimens resulting in efficacious treatments against resistant strains, and persistent infections is a priority.

A clinically relevant drug target is generally considered to be one that is essential for infection and disease progression. Rather than pursuing drugs that target metabolic pathways and macromolecular structures of current TB drugs, the research emphasis of our drug discovery program has been septum formation and cell division protein, specifically FtsZ. Chemical inhibition and molecular approaches have substantiated FtsZ as a viable drug target in *M. tuberculosis* [3]–[5]. This is consistent with the work of others that have shown that FtsZ inhibition has antimicrobial activity against *M. tuberculosis* [6], [7]. In addition to establishing FtsZ as a promising molecular target our studies characterizing the activity of albendazole and thiabendazole in *M. tuberculosis* demonstrate that benzimidazoles in general are an appropriate structural platform for TB drug discovery efforts [5]. Concordant with these biochemical studies our ongoing drug discovery consortium pursued the development of novel taxanes and benzimidazoles to treat TB infections [8], [9]. This work led to the discovery that novel trisubstituted benzimidazoles target FtsZ with a novel mode of action [4], [10], [11]. Our research efforts and the work of others have established FtsZ to be a drug target in *M. tuberculosis* [4], [10], [11]. Further substantiating FtsZ as a clinically relevant drug target and trisubstituted benzimidazoles as a drug platform is the identified broad-spectrum activity of substituted benzimidazoles against various bacterial pathogens [4], [10]–[12].

In our continued effort to find effective compounds against FtsZ, a new series of substituted benzimidazoles has been designed and synthesized based on SAR studies on 63 compounds [10], [11]. The work presented here expands on our previous reports by demonstrating the activity of the current lead compound, SB-P17G-A20, against *M. tuberculosis* H37Rv and clinical isolates and efficacy in the acute mouse model of *M. tuberculosis* infection. Time-kill curves were performed and, metabolic stability and plasma stability were determined to assess the potential *in vivo* pharmacokinetics and pharmacological performance of SB-P17G-A20. Together, these studies demonstrate that SB-P17G-A20 has potency against *M. tuberculosis* clinical strains with varying susceptibility and efficacy in animal models of infection, and that trisubstituted benzimidazoles continue to be a platform for the development of novel inhibitors with efficacy.

## Materials and Methods

### *Mycobacterium Tuberculosis* Strains, Media and Drug Conditions

The laboratory reference strain *M. tuberculosis* H37Rv was used for standard minimal inhibitory concentrations and kill characteristics analysis [13]. *M. tuberculosis* Erdman (TMCC 107) was used in the animal model of *M. tuberculosis* infection [14], [15]. Clinical isolates TN587, W210, NHN335, and NHN20 were described previously [16], [17]. For *in vitro* assays, *M. tuberculosis* was grown in Difco 7H9 Middlebrook liquid media (BD Biosciences, 271310) with 10% Middlebrook OADC Enrichment (VWR, 9000-614), 0.05% Tween (G-Biosciences, 786-519), and 0.2% Glycerol at 37°C or *M. tuberculosis* was grown on Difco Middlebrook 7H11 agar (BD Biosciences, 283810) supplemented with 10% OADC. For colony forming unit assays from animal studies the agar plates were supplemented with 1% asparagine and carbenicillin 50 mg/L (Sigma, C1389) and cycloheximide 10 mg/L (Sigma, C7698). Mutant selection studies were performed on solid medium containing drug candidate relative to experimentally determined MIC.

SAR-based drug design was used to develop a new series of 6-*N, N*-dimethylamino next generation trisubstituted benzimidazoles [10], [11]. Metronidazole (Sigma cat# M1547), rifampicin (Sigma cat# 83907), isoniazid (INH), SB-P17G-C2 and SB-P17G-A20, used in *in vitro* assays were dissolved in DMSO. Isoniazid was dissolved in water, filter sterilized and delivered IP in animal studies. The benzimidazole SB-P17G-A20 was dissolved in a 25% Solutol, 25% Ethanol in PBS diluent and delivered IP to animals.

### *In vitro* Growth Assays of Treated Bacteria

MIC values for the benzimidazole SB-P17G-A20 against *M. tuberculosis* and the clinical isolates were determined using a modified 96-well microplate Alamar Blue assay (MABA) [10], [18]. Three independent experiments were performed for the MICs and the standard error of the mean was calculated. A dose response curve with standard errors was generated for SB-P17G-A20 by graphing the log_10_ drug concentrations against the difference in growth between the treated bacteria and control bacteria from three independent experiments using GraphPad Prism Version 5.0d for Mac OS X (GraphPad Software, San Diego CA., USA, www.graphpad.com). Briefly, culture tubes were inoculated with *M. tuberculosis* H37Rv OD_600 nm_ 0.010, followed by the addition of the compound at different concentrations. The bacteria were grown at 37°C and readings were taken every 24 h for 7 days. The mean and standard error (SE) for the OD_600 nm_ values were plotted against time using GraphPad Prism Version 5.0d for Mac OS X (GraphPad Software, San Diego CA., USA, www.graphpad.com). At day 0, 2, 4 and 6 aliquots were removed from each culture, dilution series were made and plated for CFUs. The mean and SE for the CFU values were plotted against time using GraphPad Prism Version 5.0d for Mac OS X (GraphPad Software, San Diego CA., USA, www.graphpad.com). MIC values of SB-P17G-C2 against clinical isolates (0.06–0.13 µg/mL) were determined and reported [11].

### *In vitro* Combinatorial Drug Studies

A checkerboard *in vitro* 96 well plate method was used to examine possible antagonism between the SB-P17G-A20 and rifampicin. Briefly, each was diluted in a 96 well plate in a checkerboard pattern, bacteria were added and the plates were incubated at 37°C. AlamarBlue was added at day 6 and the plates were read 24 h later. The Fractional inhibitory concentration (FIC) is defined as the MIC of a drug in combination divided by the MIC of that drug alone and the fractional inhibitory index (FICI) is the sum of the FIC’s (ΣFIC) for the drugs tested in the combination [19]. When the ΣFIC is less than 0.5 the drugs are considered synergistic, when the ΣFIC is between 0.5 and 4 there is no enhanced activity, and when the ΣFIC is greater than 4 the drugs are antagonistic.

### *Mtb* FtsZ Protein Preparation

*E. coli* expression plasmid encoding the *ftsz* gene (pET 15b vector) was transformed into 100 µL of BL21(DE3) cells. The transformed cells were plated onto LB plates, containing 100 µg/mL ampicillin. The antibiotic concentration was kept the same for the following steps. The plates were incubated overnight at 37°C. The colonies were picked and grown in 10 mL of LB media at 37°C at 250 rpm shake rate. The inoculum was transferred to 1 L of LB media in a 4 L flask and grown to an OD of 0.6 at A600. Then, 1 mM IPTG was added to induce protein expression overnight at 25°C at 250 rpm shake rate. Next day the cells were harvested at 5K rpm for 15 min and re-suspended in approximately 20–30 mL binding buffer (500 mM NaCl, 20 mM sodium phosphate, pH 7.8). The re-suspended cells were lysed using cell disruptor. The lysate was centrifuged in an ultracentrifuge at 33K rpm, 4°C for 90 min. The supernatant was filtered and loaded onto a Ni^2+^-NTA column, washed with 50 mL of binding buffer and eluted using a gradient of binding buffer with 30–500 mM imidazole. The eluted protein was first dialyzed against the polymerization buffer (50 mM MES, 5 mM MgCl_2_, 50 mM KCl, pH 6.5) and then polymerization buffer containing 10% v/v glycerol. The protein was then concentrated and stored at −80°C for further use. Since the aromatic residues in *Mtb* FtsZ protein are low (Tyr: 1, Trp: 0), it is not reliable to follow the concentration of protein by scanning at A280. The concentration of protein was therefore ascertained using the Bradford kit from Sigma.

### Transmission Electron Microscopy (TEM) Analysis

A stock solution of compound SB-P17G-A20 was prepared in ethanol. *M. tuberculosis* FtsZ (5 µM) was incubated with 40 or 80 µM of compound SB-P17G-A20 in the polymerization buffer (50 mM MES, 5 mM MgCl_2_, 100 mM KCl, pH 6.5) for 15 min on ice. To each solution was added GTP to the final concentration of 25 µM. The resulting solution was incubated at 37°C for 30 min. The incubated solution was diluted 5 times with the polymerization buffer and immediately transferred to carbon coated 300 mesh formvar copper grid and negatively stained with 1% uranyl acetate. The samples were viewed with a FEI Tecnai12 BioTwinG transmission electron microscope at 80 kV. Digital images were acquired with an AMT XR-60 CCD digital camera system.

#### Plasma stability studies in human and mouse plasma

Blood was collected from animals using lithium heparin as anticoagulant, in the Animal Research & Welfare Domain at Disposition, Safety & Animal Research Montpellier (371 rue du Pr. J. Blayac, 34184 Montpellier Cedex 04, France) or vendors such as Charles River. Human plasma using lithium heparin as anticoagulant was provided by the EFS (Etablissement Français du Sang) of Montpellier, France. Plasma was spiked with the compound in order to obtain a final drug concentration of 100 ng/mL (expressed as non-salified compound). The spiked plasma (dedicated to C_1h_ and C_4h_) was then incubated at 37°C for 1 h and 4 h. Plasma samples were analyzed by LC-MS/MS following protein precipitation. The limit of quantification for compounds was 10 ng/mL. *Chromatographic conditions for LC analysis*: 10 µL of the sample was injected to Luna Phenomenex C8 (50 mm×2.0 mm, 3 µm), Solvent A: ammonium acetate (0.15 g)/formic acid (2 mL)/HPLC water up to 1000 mL. Solvent B: ammonium acetate (0.15 g)/formic acid (2 mL)/Methanol (200 mL)/Acetonitrile up to 1000 mL, flow rate of 0.3 mL/min, *t* = 0–6 min, gradient of 10–90% of B. Retention time for SB-P17G-C2 was 1.85 min and for SB-P17G-A20 was 3.3 min. *MS/MS condition used for detection:* Finnigan TSQ Quantum disco instrument, Excalibur version 2.0 acquisition system, ESI positive ion ionization mode. The mean percent of difference between C_1h_ or C_4h_ and C_0_ concentrations was calculated.

M%D (%)  = 100 × [(C_1h_ or C_4h_)–C_0_]/C_0_

The compounds were considered as stable if the mean percent of difference between C_4h_ and C_0_ concentrations was less than 20%.

#### Evaluation of oxidative metabolic lability in mouse/human liver microsomes

Microsomal fractions were prepared in the Drug Disposition Domain at DSAR Montpellier (371 rue du Pr. J. Blayac, 34184 Montpellier Cedex 04, France). SB-P17G-C2 or SB-P17G-A20 at 5 µM concentration was incubated with microsomal proteins (human or mouse, 1 mg/mL) in an incubation buffer containing phosphate 0.1 M, pH 7.4 and 1 mM NADPH as cofactor for cytochrome P-450 (CYP). The flavin-containing monooxygenases (FMO)-dependent reactions were run in the presence of bovine serum albumin (BSA, 0.1%) for the duration of 0 and 20 min (with or without microsomal proteins and/or cofactors). Enzyme activity was stopped with one volume of acetonitrile containing the internal standard, dextromethorphan. Following protein precipitation with acetonitrile and their removal by centrifugation, supernatant fluids were analyzed by LC/MS-MS.

#### Chromatographic conditions for LC analysis

SB-P17G-A20: 5 µL of the sample was injected to Hypersil Gold Thermo C18 (50 mm×2.1 mm, 1.9 µm), Solvent A: ammonium acetate (0.08 g)/formic acid (2 mL)/HPLC water up to 1000 mL. Solvent B: ammonium acetate (0.08 g)/formic acid (2 mL)/methanol (200 mL)/acetonitrile up to 1000 mL, flow rate of 0.5 mL/min, t = 0–3 min, gradient of 10–100% of B. Retention time for SB-P17G-A20 was 1.52 min.

SB-P17G-C2: 10 µL of the sample was injected to Kinetex C18 (30 mm×2.1 mm, 2.6 µC), Solvent A: HPLC-grade water up to 1000 mL, formic acid (0.1%). Solvent B: acetonitrile up to 1000 mL, formic acid (0.1%); flow rate of 0.75 mL/min, t = 0–1.5 min, gradient of 5–95% of B. Retention time for SB-P17G-C2 was 0.73 min.

#### MS/MS condition used for detection

Thermo Finnigan TSQ Quantum Ultra instrument, ESI positive ion ionization mode. Each compound is studied in duplicates. Results are expressed in percentage of lability (or total metabolism).

Total metabolism  =  [1– (UC_1_ Peak Area)/(UC_2_ Peak Area)] × 100.

UC_n_  =  Unchanged Compound in condition n;

n = 1: incubation in the presence of NADPH cofactor after T = 20 min.

n = 2: incubation without cofactor at T = 0 min.

### Modified Rapid Murine Model

Modifications were made to the short term model. Briefly, *M. tuberculosis* strain Erdman was delivered to C57BL/6-Ifngtm1ts (Jackson Laboratories, Bar Harbor, Me) by aerosol using a Middlebrook aerosol generation devise (Glas-Col, Terre Haute, IN). Treatments were given days 5 to 14 post-infection. INH was delivered IP 20 mg/kg qd and SB-P17G-A20 was delivered 50 mg/kg IP bid. Controls were infected and treated with vehicle only bid. Animals were sacrificed day 15 post-infection and the lungs and spleens were harvested. The organs were homogenized in saline, diluted, and plated. Bacterial colonies were counted, the colony counts were converted to logarithms and outliers were identified by the Grubbs’ Test using an online calculator (GraphPad Software, San Diego CA., USA www.graphpad.com). The one-way t-test at a 95% confidence interval, using GraphPad Prism Version 5.0d for Mac OS X (GraphPad Software, San Diego CA., USA www.graphpad.com), was used to compare treatment groups and infected controls, to calculate p-values, and to produce scatter plots.

### Ethics Statement

All use of vertebrate animals at Colorado State University is conducted under AAALAC approval and has an OLAW number of A3572-01. Animals are housed in a state-of-the art ABL-3 facility that is supervised by full-time staff veterinarians and a large number of support staff. The CSU animal assurance welfare number is A3572-01 under file with the NIH. Veterinary care is consistent with the recommendations of the American Veterinary Medical Association (AVMA) Guidelines.

## Results

### Optimized Compound SB-P17G-A20 has Activity against *M. Tuberculosis* Clinical Isolates and Low Spontaneous Resistant Frequency

**Figure 1:**
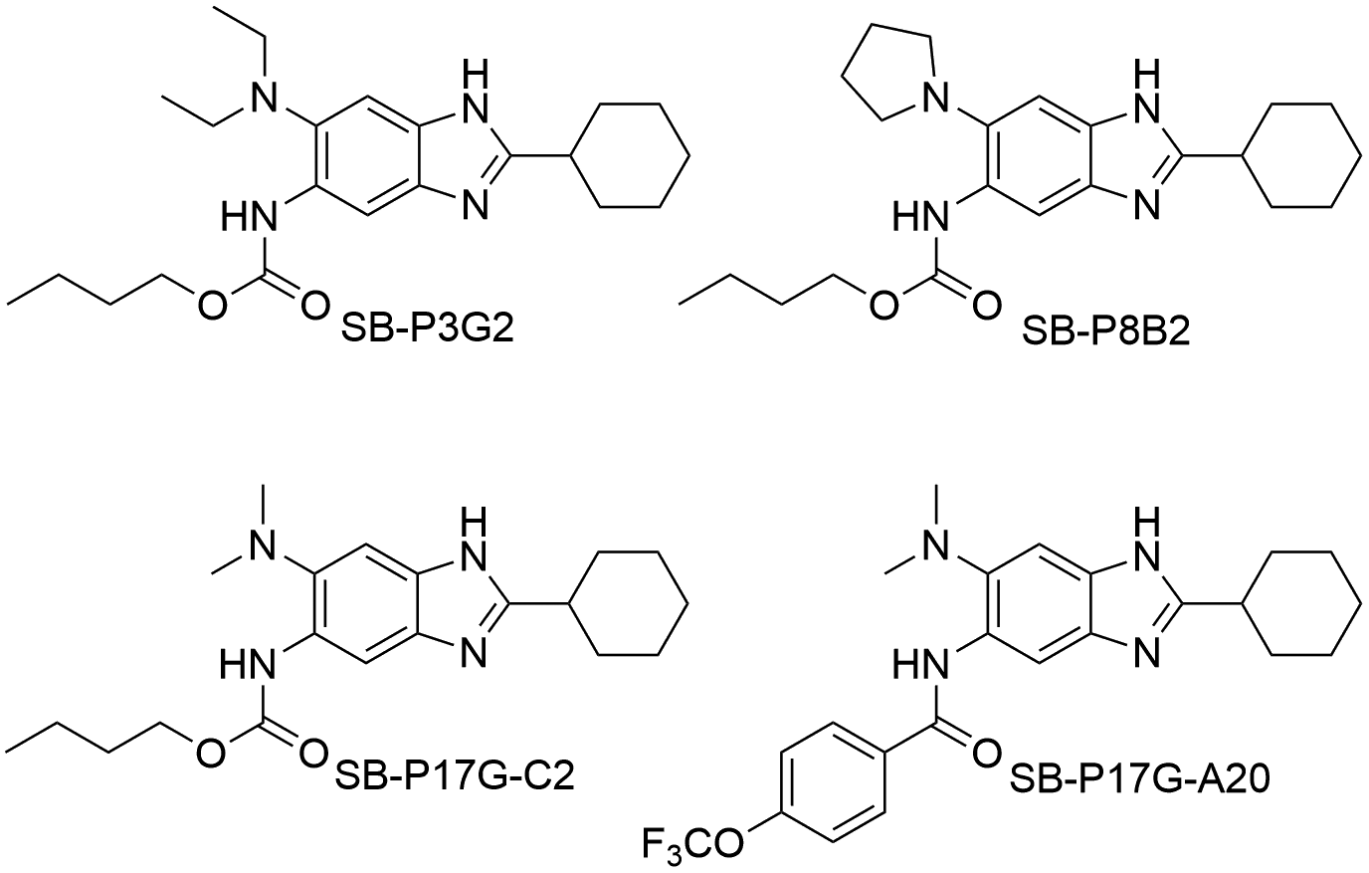
Structure of lead benzimidazoles.

**Figure 2:**
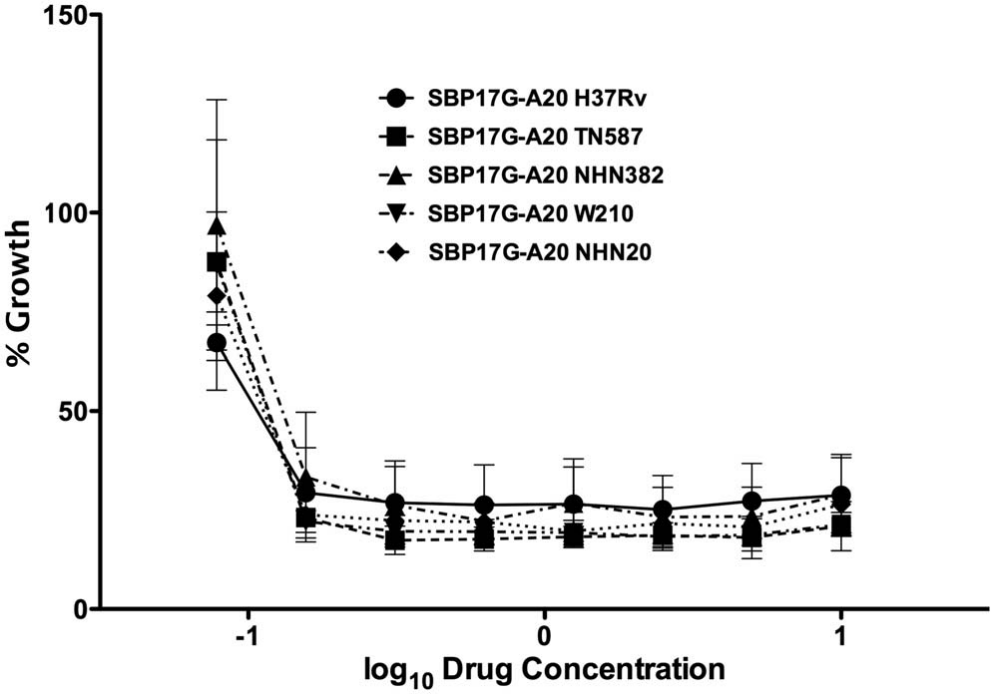
Activity of SB-P17G-A20 against *M. tuberculosis* clinical isolates. Dose response curves were generated from MABA data for *M. tuberculosis* strains (H37Rv, TN587, NHN382, W210 and NHN20) treated with SB-P17G-A20. The curves were generated by graphing the log_10_ drug concentrations against the difference in growth between the drug treated wells and control wells using GraphPad Prism Version 5.0d for Mac OS X (GraphPad Software, San Diego CA., USA, www.graphpad.com).

Previously we reported that select benzimidazoles demonstrated activity against *M. tuberculosis* H37Rv and representative clinical isolates with different susceptibilities to therapeutic tuberculosis drugs [10], [11]. Based on the SAR studies on 63 compounds, we identified several 2-cyclohexyl-5-acylamino-6-*N, N*-dimethylaminobenzimidazoles with MIC in the range of 0.06–0.63 µg/mL [11]. The two most active compounds, SB-P17G-C2 and SB-P17G-A20 (Figure 1) with MIC 0.06 and 0.16 µg/mL, respectively, were selected as leads. The MIC for SB-P17G-A20 against different clinical stains of *M. tuberculosis* is 0.16 µg/mL, demonstrating that SB-P17G-A20 is equally potent against drug-sensitive and drug-resistant strains of *M. tuberculosis* (Table 1). The growth of *M. tuberculosis* in the presence of SB-P17G-A20 was sigmoidal indicating that as the compound concentration increased the viable bacteria decreased until a concentration was reached where no additional killing was detected (Figure 2). Similarly, dose curves for the clinical isolates treated with SB-P17G-A20 were generated and superimposed on the same graph with the dose curve generated for *M. tuberculosis* H37Rv to further demonstrate that there were no differences in the kill characteristics between the different *M. tuberculosis* strains and clinical isolates. Together, the MIC values and inhibitory characteristics of SB-P17G-A20 were also similar for each strain regardless of resistance status indicating that there are no inherent cross-resistance concerns (Figure 2).

**Table 1:** Susceptibility of *M. tuberculosis* strains to SB-P17G-A20.

	*Mycobacterium tuberculosis* strains				
	H37Rv µg/mL	TN587 µg/mL	NHN382 µg/mL	W210 µg/mL	NHN20 µg/mL
SB-P17G-A20	0.16	0.16	0.16	0.16	0.16

To further assess potential drug resistance issues with the benzimidazole drug class or FtsZ inhibitors in general, *M. tuberculosis* H37Rv (2×10^9^ cells) was plated on 1.6 µg/mL of SB-P17G-A20, which is 10 times the experimentally determined MIC. This approach determines the probability that a mutation is present in the bacterial population that provides a selective advantage in the presence of the drug thereby conferring resistance. It does not account for resistance that arises due to many mutation events selected for in a single cell. No mutants where observed, and no single resistant colony was obtained.

### SB-P17G-A20 Inhibits FtsZ Polymerization

**Figure 3:**
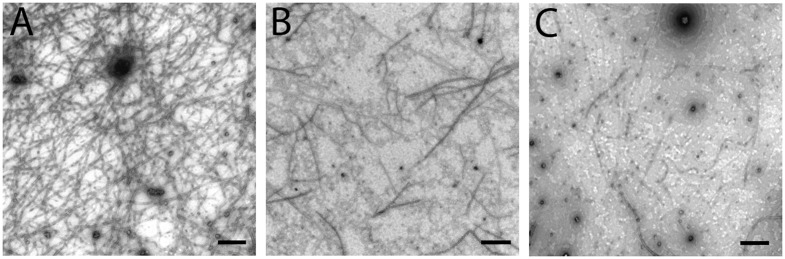
Transmission Electron Microscopy of FtsZ. FtsZ (5 µM) was polymerized by GTP (25 µM) in the absence (A) and presence of SB-P17G-A20 at 40 µM (B) and 80 µM (C). Images are at 49,000x magnification (scale bar 500 nm).

Transmission electron microscopy (TEM) imaging of *Mtb* FtsZ treated with SB-P17G-A20 demonstrated the ability of the compound to inhibit polymerization and aggregation. *Mtb* FtsZ (5 µM) treated with SB-P17G-A20 at 40 µM and 80 µM concentration following addition of GTP (25 µM) formed fewer, shorter and thinner FtsZ polymers when compared to the untreated protein. In the absence of inhibitor, *Mtb* FtsZ formed a dense network of long polymers, which tend to aggregate (Figure 3A) while in the presence of 40 µM SB-P17G-A20, the length, density and aggregation was visibly reduced (Figure 3B), and the effect is more apparent at 80 µM treatment where dispersed FtsZ polymers are observed (Figure 3C). These studies confirm that FtsZ is the molecular target of SB-P17G-A20, which is consistent with our previous report for the mode of action for substituted benzimidazoles [11].

### Plasma Stability and Metabolic Liability of SB-P17G-A20 and SB-P17G-C2

Compounds with poor plasma stability often have short t_½_ and high clearance. To assess the potential *in vivo* pharmacokinetics (PK), the stability of SB-P17G-A20 and SB-P17G-C2 were evaluated in the presence of human and mouse plasma. SB-P17G-A20 and SB-P17G-C2 were found to be stable in human plasma with only 0.1% and 6.1% hydrolysis, respectively after 4 h of incubation (Table 2). SB-P17G-C2 was highly unstable in mouse plasma being hydrolyzed 87.6% after a 4 h incubation (Table 2). In contrast, SB-P17G-A20 was found to be stable in mouse plasma with only 24.4% hydrolysis after a 4 h incubation.

**Table 2:** Plasma stability and liver microsome lability of SB-P17G-C2 and SB-P17G-A20.

Compound	Plasma Stability (% hydrolysis)		Liver Microsome Lability	
	Human (4 h)	Mouse (4 h)	Human	Mouse
SB-P17G-C2	6.1	87.6	90%	96%
SB-P17G-A20	0.1	24.4	39%	45%

Metabolic stability is also often a major limitation for lead drug candidates. To assess the extent of metabolic conversion SB-P17G-A20 and SB-P17G-C2 were evaluated using a microsomal stability assay. SB-P17G-C2 was found to be highly labile with 90% and 96% conversion in the presence of human and mouse liver microsomes, respectively (Table 2). In comparison, SB-P17G-A20 exhibited only moderate lability in the presence of liver microsomes with 39% conversion in human liver microsomes and 45% conversion in mouse liver microsomes (Table 2). These data along with previously published toxicity results [11] indicate that SB-P17G-A20 has much more favorable physiochemical properties than SB-P17G-C2 and is considered the lead benzimidazole candidate.

### Killing Characteristics of SB-P17G-A20 against Whole Bacteria

**Figure 4:**
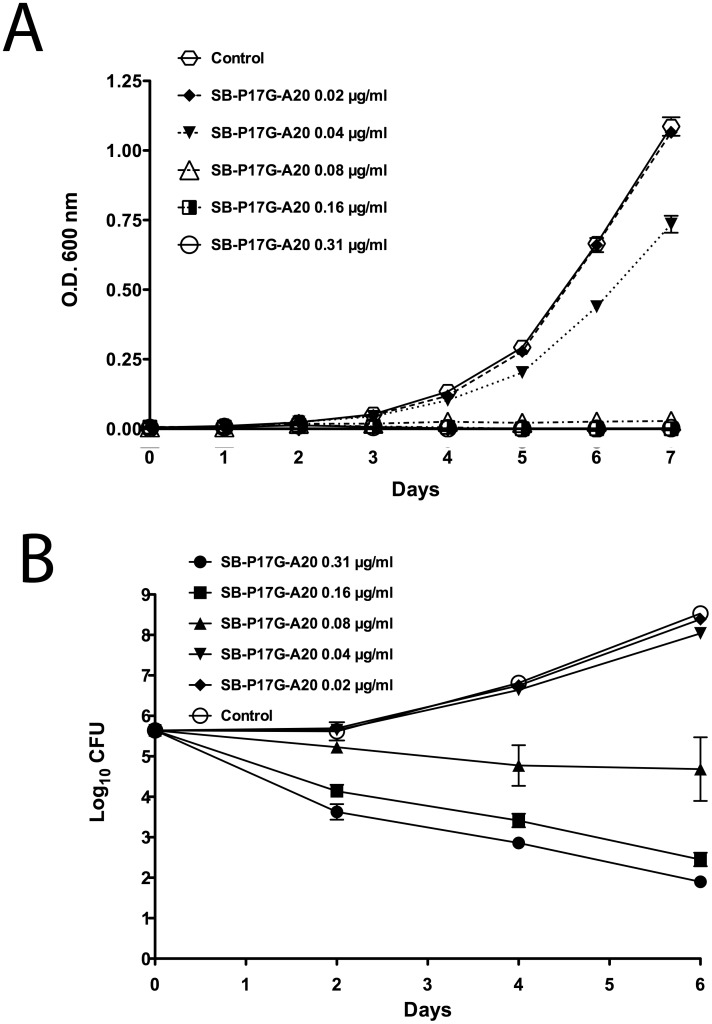
Killing characteristics of SB-P17G -**A20 against whole bacteria.** The time dose curves were generated from OD_600 nm_ (a) and from CFU enumeration (b) data. Different concentrations of the compound were tested in triplicate and the mean and standard deviation of the OD_600 nm_ values or the CFU counts from Day 0, 2, 4, and 6 were plotted against time using GraphPad Prism Version 5.0d for Mac OS X (GraphPad Software, San Diego CA., USA, www.graphpad.com).

To investigate the killing characteristics of SB-P17G-A20, bacterial growth in the presence of different concentrations of the compound was monitored by OD600 nm and bactericidal effect was monitored over 7 days (Figure 4A, B). The growth curve of *M. tuberculosis* in the presence of various concentration of SB-P17G-A20 showed that this is a concentration dependent inhibitory agent. *M*. *tuberculosis* did not grow in the presence of SB-P17G-A20 at concentrations near the MIC. Notably, bacterial growth was affected by sub-MIC concentrations ranging from 0.16 µg/mL to 0.02 µg/mL (Figure 4A). Similarly, the viability of *M. tuberculosis* as determined by plating and outgrowth is also reduced at concentrations of SB-P17G-A20 below the MIC. SB-P17G-A20 at 0.08 µg/mL steadily reduced bacterial viability with concentrations of 0.16 µg/mL to greater than the MIC having an increased impact (Figure 4B). Together, these data indicate that the FtsZ cell division protein inhibitor SB-P17G-A20 is a concentration-dependent inhibitor with sub-MIC inhibitory characteristics.

### SB-P17G-A20 is not Antagonistic with the First Line Antitubercular Rifampicin

As part of our TB drug discovery program we assessed the combinatorial activity of lead drug candidates and a selected front-line TB drug. We evaluated SB-P17G-A20 in the presence of rifampicin and found that SB-P17G-A20 activity against *M. tuberculosis* was enhanced 2–4 fold in the presence of rifampicin and rifampicin activity against *M. tuberculosis* was enhanced 2 fold in the presence of SB-P17G-A20. The resulting ΣFIC for rifampicin in combination with SB-P17G-A20 was 0.75 indicating that these drugs are not antagonistic and therefore could be used in combination to treat a TB infection. This result along with our previous results confirm that benzimidazoles in general are not antagonistic with one of the front-line clinical drugs and, in fact enhance the activity of rifampicin against *M. tuberculosis* between by 2 to 4-fold.

### SB-P17G-A20 Demonstrates Efficacy in a Tuberculosis Murine Model of Infection

**Figure 5:**
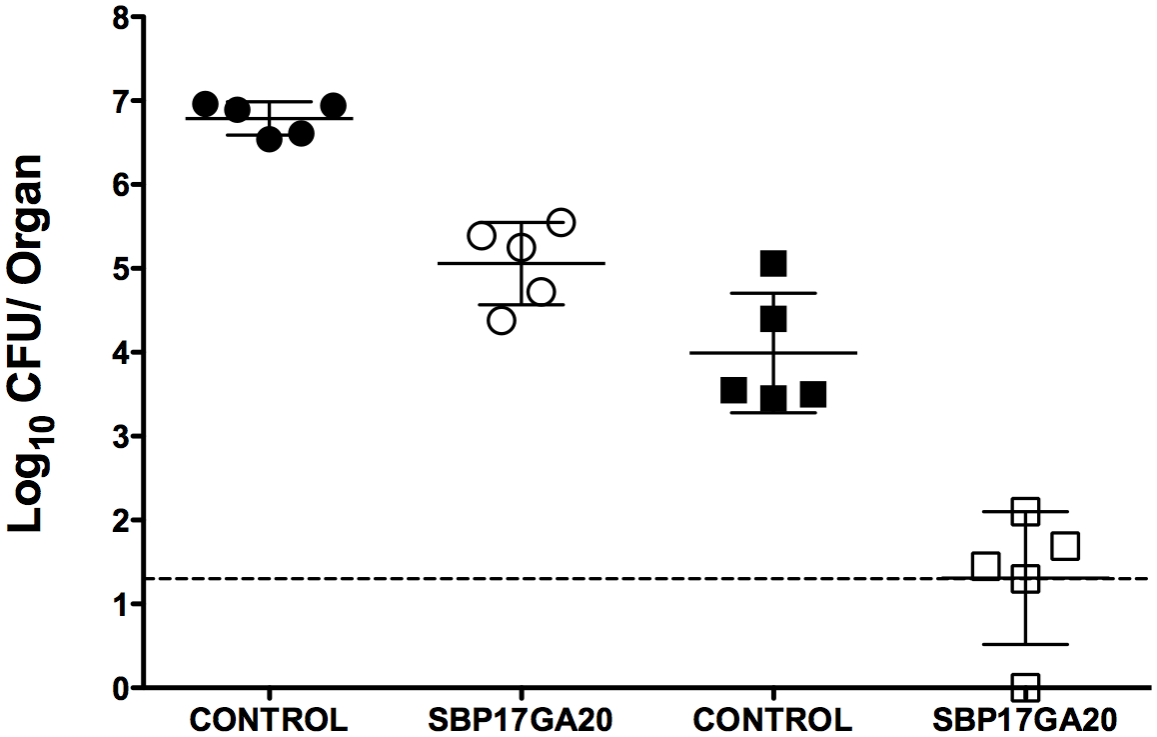
Efficacy of SB-P17G-A20 in a tuberculosis murine model of infection. Scatter plot of the CFU counts from the lung and spleens of infected mice after drug therapy with SB-P17G-A20 delivered IP at 50 mg/kg bid. The colony counts were converted to logarithms. The lower level of detection was 1 log_10_ CFU. Outliers were identified by the Grubbs’ Test using an online calculator. (GraphPad Software, San Diego CA., USA www.graphpad.com). A scatter plot of the CFU data from the lung and spleen of individual mice from the treatment and control groups were plotted with the mean and SE from each group using GraphPad Prism Version 5.0d for Mac OS X (GraphPad Software, San Diego CA., USA www.graphpad.com).

To assess the efficacy of SB-P17G-A20, it was delivered 50 mg/kg IP bid in a rapid acute murine model of infection (Figure 5). INH was delivered IP 20 mg/kg qd as a control and reduced the CFU counts in the lung and spleen below the level of detection for this experiment. In this acute model, all mice treated with SB-P17G-A20 had bacterial counts in the lung less than untreated infected controls resulting in a reduction in the bacterial load in the lungs of 1.73±0.24 log_10_ CFU (p value<0.0001). Similarly, all treated mice with SB-P17G-A20 had bacterial counts in the spleen lower than the untreated infected controls, and 1 mouse had no detectable bacteria at the lowest level of detection, resulting in a reduction of 2.68±0.48 log_10_ CFU (p value 0.0002) bacterial load in the spleen. However, when SB-P17G-C2 was assessed for efficacy, there was no significant reduction in the bacterial load in the lungs or spleen (data not shown). This is consistent with the plasma and metabolic stability of SB-P17G-A20 and SB-P17G-C2. It is important to note that SB-P17G-A20 is the first lead compound in this class that has significantly reduced the bacterial load in both the lung and spleen.

## Discussion

*M. tuberculosis* continues to be one of the leading causes of death due to an infectious disease. The emergence of *M. tuberculosis* strains that are resistant to frontline TB drugs and therefore TB drug combinations has hampered the management and control of this disease. SB-P3G2 was well-characterized and was shown to inhibit FtsZ polymerization in a dose dependent manner. Since the compounds, SB-P3G2 and SB-P8B2 have shown promising antibacterial activities *in vitro* and *in vivo*, we continued optimization of substituted benzimidazoles through systematic structural modifications based on SAR studies. This resulted in the development of a series of 2-cyclohexyl-5-acylamiono-6-*N, N*-dimethylaminobenzimidazoles with MICs in the range of 0.06–0.63 µg/mL against *M. tuberculosis* and clinical isolates with different resistance profiles [11]. From this series, SB-P17G-C2 and SB-P17G-A20 where identified as interesting lead compounds with MIC values of 0.06 µg/mL and 0.16 µg/mL, respectively.

SB-P17G-A20 has activity against *M. tuberculosis* H37Rv and clinical isolates with different resistance profiles, which is consistent with our previous results with this structural class of compounds, such as SB-P17G-C2 [11]. SB-P17G-A20 is equally effective against *M. tuberculosis* and clinical isolates over a wide concentration range. This is important because it shows that SB-P17G-A20 is bactericidal against existing clinical strains and at pharmacologically achievable concentrations. Notably, it was observed that in some cases bacterial growth was reduced by SB-P17G-A20 at concentrations as low as 0.125 X MIC. To evaluate the potential use of SB-P17G-A20 in combination with frontline clinical drugs, SB-P17G-A20 was tested in combination with rifampicin. This revealed that the activity of SB-P17G-A20 was enhanced by the presence of rifampicin, while it enhanced the activity of rifampicin, thus allowing these two drug classes to be used in combination.

We were unable to select for SB-P17G-A20 spontaneous resistant mutants of *M. tuberculosis*. Development of resistant mutants was attempted by independent selection and growth of *M. tuberculosis* H37Rv. As *M. tuberculosis* exhibits low genetic diversity in general, this result is not surprising. In addition, the inability to derive high-level resistant mutants is consistent with our previous molecular studies with dominant-negative temperature sensitive FtsZ merodiploid strains of *M. tuberculosis* [3]. These studies demonstrated that mutations in FtsZ resulted in changes in protein structure and GTPase activity, which adversely affected FtsZ polymerization resulting in the dominant-negative phenotype. The observed dominant-negative phenotype did not require a large number of inactive temperature sensitive FtsZ proteins. Rather, only a few inactive FtsZ proteins can result in molecular poisoning because of the fact that FtsZ must undergo a successful polymerization event to perform its structural role. Accordingly, the failure to select for high-level resistance to SB-P17G-A20 can be attributed to the inability of FtsZ to tolerate structural changes or amino acid changes in the GTPase domain.

Because unstable compounds have short t_½_ and high clearance, and therefore poor pharmacological performance, it was necessary to determine the *in vivo* pharmacokinetic properties of SB-P17G-C2 and SB-P17G-A20 by assessing plasma stability and metabolic stability in liver microsomes. While both compounds were stable in human plasma studies using mouse plasma revealed that 90% of SB-P17G-C2 was hydrolyzed in 4 h. The significant difference in plasma stability results from the carbamate moiety at the 5-position of SB-P17G-C2, which is an amide group in SB-P17G-A20 that is more resistant to hydrolysis. Similarly, the conversion rate of SB-P17G-A20 was significantly slower than SB-P17G-C2 in the presence of liver microsomes. These data indicate that SB-P17G-A20 has much better *in vivo* pharmacokinetic properties than SB-P17G-C2.

Based on the potency and the kill-curve characteristics, along with the stability data, SB-P17G-A20 was advanced to efficacy studies using an acute mouse model of infection to assess its potential as a lead compound. In this model SB-P17G-A20 significantly reduced the bacterial load in both the lungs and the spleen. In particular, SB-P17G-A20 either killed the bacteria upon dissemination or within the spleen because the bacteria recovered from the spleen following treatment where at the lowest level of detection if detectable at all. SB-P17G-C2 was also evaluated for efficacy in an acute mouse model of infection. However, this compound did not significantly reduce the bacterial load in the lungs or spleen, which is attributed to its poor stability in mouse plasma and microsomes. Thus far, SB-P17G-A20 is the most potent trisubstituted benzimidazole developed and tested in the animal model of infection as determined by the overall reduction in the bacterial load in the lungs and spleen.

The optimized substituted benzimidazole, SB-P17G-A20, was characterized *in vitro* and *in vivo* for potency against *M. tuberculosis* clinical strains and efficacy in a *M. tuberculosis* murine model of acute infection. *In vitro* studies have revealed that SB-P17G-A20 has activity against strains with various drug susceptibility profiles in a concentration-dependent manner, and possesses bactericidal activity at sub-MIC concentrations. In addition, SB-P17G-A20 is not antagonistic to the front-line TB drug rifampicin, providing the opportunity to be used in combination therapies. SB-P17G-A20 showed much better plasma/metabolic stability than other lead candidates and although the efficacy of SB-P17G-A20 in a chronic model of infection has not been performed yet, the demonstrated efficacy in an acute infection model substantiates that the 2, 5, 6-trisubstituted benzimidazole scaffold is a platform for the discovery and development of anti-tubercular drugs.
